# The effects of *Ostertagia occidentalis *somatic antigens on ovine TLR2 and TLR4 expression

**Published:** 2015

**Authors:** Hassan BORJI, Alireza HAGHPARAST, Nooshinmehr SOLEIMANI, Mohammad AZIZZADEH, Mohammad Hossein NAZEMSHIRAZI

**Affiliations:** 1 *Dept. of Pathobiology, School of Veterinary Medicine, Ferdowsi University of Mashhad, Mashhad, Iran*; 2 *Dept. of Clinical Science, School of Veterinary Medicine, Ferdowsi University of Mashhad, Mashhad, Iran*; 3 *Central Laboratories of Khorasan Razavi Veterinary Organization, Mashhad, Iran*

**Keywords:** *Ostertagia occidentalis*, Peripheral blood mononuclear cells, Toll-like receptors

## Abstract

***Background:*** Recognition of helminth-derived pathogen associated molecular patterns (PAMPs) by pattern recognition receptors (PRRs), including toll like receptors (TLRs) is the first step towards initiating anti–helminth immune responses.

***Methods*** **:** Using somatic antigens of* Ostertagia occidentalis, *an important abomasal parasite of ruminants, the expression of ovine TLR2 and TLR4 in peripheral blood mononuclear cells (PBMCs) was analyzed by real-time quatitative reverse-transcription polymerase chain reaction (qRT-PCR). Somatic antigens of *O. occidentalis* were prepared to stimulate ovine PBMCs in a time and dose dependent manner.

***Results*** **:** A high expression of TLR2 and TLR4 was observed in PBMCs cultured with somatic antigens of the parasites specially when PBMCs were cultured with 100 µg/ml of somatic antigens and incubated for 2h. Up-regulation of TLR2 expression was more pronounced and evident in our study.

***Conclsusion*** **: **Somatic antigens of *O. occidentalis* have immunostimulatory and dominant role on peripheral immune cells. This study provide for the first time evidence of induction of TLRs in ovine PBMCs by somatic antigen of *O. occidentalis*

## Introduction

Ostertagia occidentalis is the most economically ovine parasitic gast-roenteritis in temperate regions of the world. Adult worms develop in the abomasum and are approximately 1-2 cm in length when mature. They are dioescious with eggs passing out of the host in faeces and developing to infective larvae on the pasture. Infective larvae (L3) penetrate abomasal glands within 24 h of infection and grow rapidly, undergoing two moults before emerging into the lumen approximately 10 days post-infection. The brownish worms infect the gastric glands of the stomach and lead to weight loss, decreased wool production and death (1, 2). 

Anthelmintic resistance in *Ostertagia *spp to all the major anthelmintic classes is common worldwide often leading to failure of treatment and control (3, 4). This, in combination with increasing public pressure to ensure that animals and derived products are free of contaminating residues including anthelmintics, has driven the search for alternative control measures. Vaccination offers an alternative approach to drug-based control and a great deal of investment has gone into the investigation of protective antigens for this species. However, attempts at vaccination are hindered by a lack of understanding of how best to promote protective immunity to this species. 

A number of studies have investigated the immunological basis of resistance to *Ostertagia *spp. Expression of resistance to *Ostertagia *spp*. *involves changes in the abomasal microenvironment that affect the parasite in a number of ways: these include immune exclusion of incoming L3, slowing of larval development and effects on adult worm length and fecundity (5, 6). 

The type of adaptive immune response generated following either infection or immunization is influenced by the Toll-like receptor (TLR) family and associated signaling pathways (7). TLRs are the most studied and best characterized pattern recognition receptors (PPRs) which are responsible for sensing pathogen associated molecular patterns (PAMPs) and damage associated molecular patterns (DAMPs). Although the primary function of TLRs is to detect PAMPs and DAMPs and activate innate immunity, they also play an important role in the initiation and development of adaptive immune responses (8, 9). There are 11 known mammalian TLRs that recognize bacterial, viral and fungal pathogen-derived antigens. They are expressed by various cells of the immune system and epithelial cells of different organs. Many of the TLRs like TLR2 and TLR4 are present on the cell surface and a number of TLRs are located in endosomal compartment of cells, providing a broad recognition barrier for both extracellular and intracellular pathogens. Activation of this pathway in turn leads to highly coordinated expression of a number of cytokines and chemokines that control the Th2 nature of the response.

 Recognition of parasite structures is crucial for the initiation of appropriate immune responses that will allow the parasite to modulate the immune system and survive in its host. Such recognition is thought to occur through interaction of parasite derived PAMPs with pattern recognition receptors, including TLRs. The type of parasitic compounds and the PRRs involved in their recognition will determine largely the nature of the polarization and resolution of the immune responses (9). TLRs are key players in the innate immune system, but there is limited information on their involvement in innate resistance against *O. occidentalis *infection. 

*O. occidentalis *secretes and exposes numerous immunological molecules to the host immune system. By exploring the structures of these molecules we can understand the mechanisms that* O. occidentalis *uses for persisting in the host. Recent application of modern molecular and immunological approaches has revealed insights on the nature of immune responses generated during the course of *O. occidentalis *infection, although many aspects of the *O. occidentalis -*host interplay remain unexplored. 

Because of the nature of *O. occidentalis*, TLRs expressed on cell surface are likely to be involved in the recognition of the PAMPs derived parasites, and among them, TLR2 and TLR4 are the best candidates to recognize *O. occidentalis *antigens. Therefore, in the present study, we investigated the *in vitro* effect of somatic derived antigens of *O. occidentalis *on the activation and expression of ovine TLR2 and TLR4 in peripheral blood mononuclear cells.

## Materials and Methods

***Extraction of O. occidentalis somatic antigens***

The abomasum was removed from sheep challenged with *Ostertagia* (slaughtered at abattoirs in Mashhad, Khorasan-Razavi province, Iran), washed with PBS, the contents discharged and washed several times with PBS and *O. occidentalis *worms separated by filtration and microscopy. The freeze/thaw process was performed on helminths and then homogenized in glass homogenizer with an equal volume of PBS. The preparation was then disrupted by sonication in a 150 W ultrasonic disintegrator (10s on 5s off) on ice for 25 min. The supernatant was collected and centrifuged at 1500g for 15 min and the supernatant was collected as *O. occidentalis *antigens. Antigens were kept at -20 C until used. 

The concentration of antigens used in this study was measured by standard Bradford method and the integrity of their protein contents was analysed by SDS-PAGE (data not shown). 

***Isolation of PBMCs and stimulation with*** ***O. occidentalis derived*** ***antigens***

Peripheral Blood Mononuclear Cells (PBMCs) from five healthy one-month-old young lambs were collected in sterile venipucture tubes containing EDTA as anticoagulant and transferred to Immunology lab of Faculty of Veterinary Medicine. After isolating PBMCs with standard Ficoll-hypaque method, the cell viability was measured by Trypan blue staining. Then the PBMCs were placed in 6-well cell culture plates (12 x10^6^ cells/well) and cultured in RPMI1640 (Biosera, Boussens, France) containing Hepes (25mM), L-Glutamin (2mM), 10%FBS, Penicillin (100 unit/ml) and Streptomycin (100 µg/ml). Upon culture, the PBMCs were stimulated immediately with different concentrations of *O. occidentalis *derived antigens in a dose and time dependent manner (50 µg/ml and 100 µg/ml and incubated for 2h and 18h). During the culture period the plates were kept at 37C with 95% humidity and 5% CO_2 .._

***RNA isolation and cDNA synthesis***

Total RNA was extracted from the cell pellets using high pure RNA isolation kit (Roche Applied Science, Penzberg, Germany) based on the manufacturer’s instructions. Then RNA integrity was tested by running the respective RNA preparations on denaturing agarose gel and observing the intensity and sharpness of 18s and 28s rRNA bands. The quantity and purity of the RNA samples were determined by measuring the absorbance at 260 and 280 nm and the A260/A280 ratio on a nanodrop 2000 spectrophotometer (Thermo Fisher Scientific, Wilmington, USA). For cDNA synthesis, 2μg of total RNA was mixed with 1μl of oligo (dT)_12-18_ primer ( Fermentas, Vilnius, Lithuania) and was diluted to 13μl with DEPC treated water, heated to 70 °C for 5 min, and then rapidly chilled on ice. Then 2μl of 10mM dNTP mix (Cinagen, Karaj, Iran) plus 4μl of 5X buffer was added and the mixture was incubated for 5 min at 37 °C. Finally, to each reaction 1μl (200 units) M-MuLV reverse transcriptase (Fermentas, Vilnius, Lithuania) was added and the mixture was incubated for 60 min at 42 °C and at 72 °C for 10 min to stop the reaction. All incubations were carried out using Thermal Cycler apparatus (Primus 25, Germany). A non-RT control was also prepared for each sample. The cDNA product was kept at -20 °C until used.

***Real-time quatitative reverse-transcription polymerase chain reaction (qRT-PCR)***

Expression of TLR2 and TLR2 was analyzed by qRT-PCR using the Corbett 6 apparatus (Rotor-Gene, Australia). Exon specific primer sequences for TLR2 and TLR4 were synthesized by Bioneer (Daejeon, South Korea) as follows: TLR2 forward: 5'-GGTTTTAAGGCAGAATCGTTTG-3', TLR2 reverse: 5'- AAGGCACTGGGTTAAACTGTG-T-3', TLR4 forward: 5'- CTTGCGTACAGGTTGTTCCTAA-3', TLR4 reverse: 5'- CTGGGAAGCTGGAGAAGTTATG-3'; and GAPDH (as internal control) forward: 5'- TCAAGAAGGT-GGTGAAGCAG-3', GAPDH reverse: 5'- TGTCGTACCAGG AAATGAGC-3'. The specificity of the amplification reaction was determined with a melting curve analysis, including fluorescence measurement every 15 seconds, 0.3 degree of temperature rise from 65 to 95°C. We performed relative quantification through normalizing the TLR2 and TLR4 genes signals with glyceraldehyde-3-phosphate dehydrogenase (GAPDH) signal. Reactions were performed in duplicate in a 20 μl volume, including 400 ng cDNA, 10 μl **S**YBRGreen PCR mastermix (QIAGEN, Germany), nuclease free water, 2pmol forward and reverse primers. For each cDNA sample, a non-RT control was also run in parallel. qPCR products were visualized by 2% agarose gel electrophoresis stained with ethidium bromide. Relative fold change expression of genes was calculated using the Pfaffl method (8). To quantify the results, a 10-fold serial dilution standard curve of a pooled of five cDNA samples obtained from PBMCs of one month old healthy young lambs as calibrator for any primer (TLR2, TLR4 and GAPDH) was performed. The same cDNA sample was used for all the standard-curve runs. A threshold of detection was set based on the duplicate control samples lacking a template. Amplification efficiency of a reaction was calculated using data collected from a standard curve with the following formula (10):

Efficiency of reaction=10-1/slope-1

***Statistical analysis***

SPSS software, version 16 (SPSS Inc., Chicago, IL, USA), was used to conduct the statistical analysis. Expressions of TLR2 and TLR4 in all groups were screened for normality using Shapiro-wilk test. The distributions were normal. A one-sample t test was used to test if the antigen could change the gene expression. 

Because of unequal variance (heteroscedasticity), comparison of expression of TLR2 and TLR4 between groups (different antigen concentration and different incubation time) was performed by kruskal wallis test. *P*<0.05 was considered as significant.

## Results

**Fig.1:**
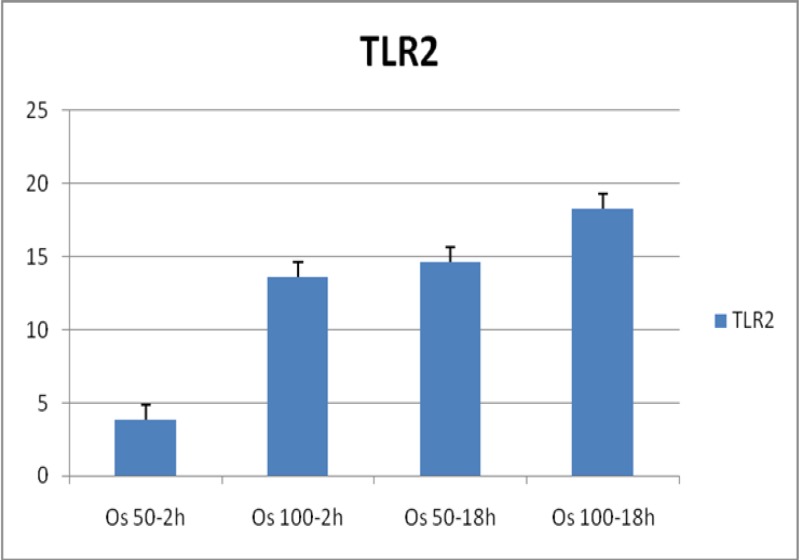
Expression of ovine TLR2 in PBMCs stimu lated with somatic antigens of *O.occidentalis *in different concentrations and time

**Fig. 2:**
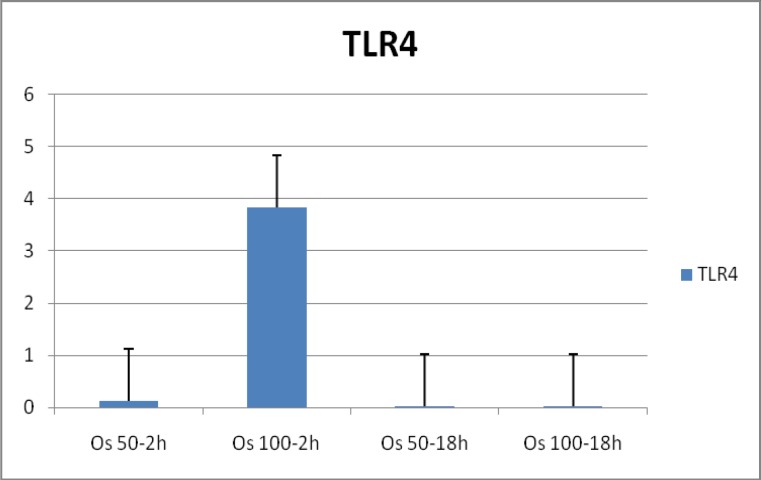
Expression of ovine TLR4 in PBMCs stimulated with somatic antigens of *O. occidentalis *in different concentrations and time

In order to evaluate the expression of TLR2 and TLR4, PBMCs obtained from one month healthy young lambs were cultured with 50 μg/ml and 100 μg/ml of somatic antigens and incubated for 2h and 18h. After isolating RNA from the cell culture pellet, qRT-PCR was performed to measure the relative expression level of ovine TLR2 and TLR4. Results showed a nearly 19 fold increase in the expression of TLR2 in nearly all time and dose dependent culture condition (Fig.1). Moreover, a 4 fold increases in the expression of TLR4 in PBMCs cultured with various concentrations of somatic antigens was observed (Fig. 2). This finding was more evident in a culture condition of 100 μg/ml of antigens and 2 hours of incubation (Table 1).

**Table 1:** Concentration of *Ostertagia occidentalis *somatic antigens on TLR2 and TLR4 expression after 2 and 18 hours

***O. occidentalis *** **somatic Ag**	**TLR2**	**TLR4**
50,2h	3.83	0.129
100,2h	13.58	3.84
50,18h	14.62	0.028
100,18h	18.25	0.022

## Discussion

Understanding how the immune system behaves towards *O. occidentalis *is a major challenge. An understanding of the biological events occurring during infection is necessary to reveal the diverse immune responses to which the parasite subjects the host and to define diagnostic, therapeutic and preventive strategies. Although many helminth antigens have been identified, our knowledge of the host immune receptors responsible for their recognition, binding and internalization is more limited. The expression profile of TLRs and their potential contribution to immune responses in ostertagiosis are poorly understood. We therefore analyzed the effects of *O. occidentalis *somatic antigens on the host innate immune responses by studying early events of activation of PBMCs to analyze its possible involvement in the mechanisms of host defense and/or parasite evasion.

 Our results indicate up-regulation of TLR2 and TLR4 by PBMCs upon exposure to somatic antigens. To our knowledge, this work evaluates for the first time the effect of *O. occidentalis *somatic antigens on TLRs expression. Our in vitro experiment correlated to the previously described study showing that TLR2 and TLR4 increased by soluble extracts from *Schistosoma mansoni* eggs (11). The increase in the expression of TLR2 and TLR4 has been also reported in stimulation of human cholangiocytes by *Cryptosporidium parvum *(12). In addition, increased levels of TLR2 and TLR4 mRNA expression were observed *in vivo* experiment in the chronic phase patients infected with cystic echinococcosis (13). In murine hymenolepidosis, levels of TLR2 and TLR4 mRNA expression have been up regulated in infected rats (14). Our observation on the increased expression of TLR2 and TLR4 suggests the involvement of these TLRs in the recognition of *O. occidentalis *pathogen-associated molecular patterns (PAMPs). TLR2 and TLR4 in immune cells recognize lipopeptide and lipoplysachharide structures from pathogens, respectively (15), but the putative ligand for these TLRs in the context of *O. occidentalis *antigens remains unknown. It might be possible that such structures are present in the somatic antigens. The increased TLR expression noticed in the present study may have been induced due to the presence of heat shock proteins in somatic antigens of* O. occidentalis*. *T. gondii*-derived heat shock protein 70 (rTgHSP70) signaling mechanisms were depended on TLR2, myeloid differentiation factor 88 (MyD88) and IL-1R-associated kinase (IRAK4), but not on TLR4 (16). 

TLR activation by parasite molecules can also trigger the expression of proinflammatory cytokine genes.TLR4 is required for Th2 immunity in response to pulmonary allergy and cross-talk between Th1 and Th2 immunity, and TLR 2 signaling induces Th2 cytokines such as interleukin IL 4, IL 5 and IL13 (8). The increased expression of TLR2 and TLR4 to the antigen surface of *O. occidentalis *may contribute to the initiation of immune responses, including Th2-cell responses that generally result in eosinophilia, goblet and mucosal mast-cell hyperplasia, which lead to the elimination of the parasite (17). Taken together, it seems likely that acute or chronic infections, as observed with helminths, are associated with modulation of innate immune receptors and such regulation has a crucial role in immune protection or immune-mediated pathology.

Recent reports on expression of TLRs in epithelial cell lines (18) are paving a way for understanding a novel regulatory function at this frontier. The general consensus emerging from these initial studies is that normal epithelium has very low expression of these receptors which makes the mucosal immune response tolerant of commensal antigens, whereas pathological conditions such as a very high antigenic dose or presence of regulatory cytokines might stimulate expression of the Toll receptor, leading to amplified innate immune response characterized by secretion of proinflammatory cytokines with consequent elimination of pathogens and probable mucosal destruction. TLRs also play a role in maintaining epithelial barrier function in response to enteric pathogens and parasites (19). Moreover, it is important to be aware that greater abomasal permeability is a TLR4 dependent process (20). Perhaps in response to the antigen surface of *O. occidentalis*, TLR4 has been implicated in the integrity of abomasal epithelium. 

## Conclusion

Our data extend the available knowledge about *O. occidentalis *somatic antigen effects on immune defenses of host. Assuming that our *in vitro* results represent, at least in part, what actually happens with PBMCs upon infection with *O. occidentalis*. The alteration in the level of expression of TLR2 and TLR4 may point the role of the innate immune system in the pathogenesis of this infection. Study of Toll receptors and parasitic molecular patterns thus offers an exciting area to unravel the mysteries of host-parasite interactions. Further investigation will be necessary to identify how such mechanisms would operate *in vivo.*

## References

[1] Simpson HV (2000). Pathophysiology of abomasal parasitism: is the host or parasite responsible?. Vet J.

[2] Simpson HV, Przemeck SM, Scott I, Thomas DG, Green RS, Reynolds GW (2009). Pathophysiology in Teladorsagia circumcincta-infected sheep selected for high fleece weight. Vet Parasitol.

[3] Shayan P, Eslami A, Borji H (2007). Innovative restriction site created PCR-RFLP for detection of benzimidazole resistance in Teladorsagia circumcincta. Parasitol Res.

[4] Papadopoulos E, Gallidis E, Ptochos S (2012). Anthelmintic resistance in sheep in Europe: a selected review. Vet Parasitol.

[5] Beraldi D, Craig BH, Bishop SC, Hopkins J, Pemberton JM (2008). Phenotypic analysis of host-parasite interactions in lambs infected with Teladorsagia circumcincta. Int J Parasitol.

[6] McNeilly TN, Devaney E, Matthews JB (2009). Teladorsagia circumcincta in the sheep abomasum: defining the role of dendritic cells in T cell regulation and protective immunity. Parasite Immunol.

[7] McGuinness DH, Dehal PK, Pleass R J (2003). Pattern recognition molecules and innate immunity to parasites. Trends Parasitol.

[8] Iwasaki A, Medzhitov R (2004). Toll-like receptor control of the adaptive immune responses. Nat Immunol.

[9] Kawai T, Akira S (2010). The role of pattern-recognition receptors in innate immunity: update on Toll-like receptors. Nat Immunol.

[10] Pfaffl MW (2001). A new mathematical model for relative quantification in real-time RT-PCR. Nucleic Acids Res.

[11] Kane CM, Jung E, Pearce EJ (2008). Schistosoma mansoni egg antigen-mediated modulation of Toll-like receptor (TLR)-induced activation occurs independently of TLR2, TLR4, and MyD88. Infect Immun.

[12] Chen XM, O’Hara SP, Nelson JB, Splinter PL, Small AJ, Tietz PS, Limper AH, LaRusso NF (2005). Multiple TLRs are expressed in human cholangiocytes and mediate host epithelial defense responses to Cryptosporidium parvum via activation of NF-kappaB. J Immunol.

[13] Shan JY, Ji WZ, Li HT, Tuxun T, Lin RY, Wen H (2011). TLR2 and TLR4 expression in peripheral blood mononuclear cells of patients with chronic cystic echinococcosis and its relationship with IL-10. Parasite Immun.

[14] Kosik-Bogacka DI, Wojtkowiak-Giera A, Kolasa A, Czernomysy-Furowicz D, Lanocha N, Wandurska-Nowak E, Salamatin R, Jag-odzinski PP (2013). Hymenolepis diminuta: analysis of the expression of Toll-like receptor genes (TLR2 and TLR4) in the small and large intestines of rats. Part II. Exp Parasitol.

[15] Van Riet E, Everts B, Retra K (2009). Combined TLR2 and TLR4 ligation in the context of bacterial or helminth extracts in human monocyte derived dendritic cells: molecular correlates for Th1/Th2 polarization. BMC Immunol.

[16] Mun HS, Aosai F, Norose K, Piao LX, Fang H, Akira S, Yano A (2005). Toll-like receptor 4 mediates tolerance in macrophages stimulated with Toxoplasma gondii-derived heat shock protein 70. Infect Immun.

[17] Seo M, Guk SM, Han ET, Chai JY (2003). Role of intestinal goblet cells in the expulsion of Gymnophalloides seoi from mice. J Parasitol.

[18] Cario E, Rosenberg IM, Brandwein SL, Beck PL, Reinecker HC, Podolsky DK (2000). Lipopolysaccharide activates distinct signaling pathways in intestinal epithelial cell lines expressing TLRs. J Immunol.

[19] Moncada DM, Kammanadiminti SJ, Chadee K (2003). Mucin and Toll-like receptors in host defense against intestinal parasites. Trends Parasitol.

[20] Peterson CY, Costantini TW, Loomis WH, Putnam JG, Wolf P, Bansal V, Eliceiri BP, Baird A, Coimbra R (2010). Toll-like receptor-4 mediates intestinal barrier breakdown after thermal injury. Surg Infect.

